# *Klebsiella pneumoniae*-OMVs activate death-signaling pathways in Human Bronchial Epithelial Host Cells (BEAS-2B)

**DOI:** 10.1016/j.heliyon.2024.e29017

**Published:** 2024-04-13

**Authors:** Federica Dell'Annunziata, Elena Ciaglia, Veronica Folliero, Valentina Lopardo, Anna Maciag, Massimiliano Galdiero, Annibale Alessandro Puca, Gianluigi Franci

**Affiliations:** a Department of Medicine, Surgery and Dentistry “Scuola Medica Salernitana", University of Salerno, 84081, Baronissi, Salerno, Italy; b Department of Experimental Medicine, University of Campania Luigi Vanvitelli, 80138, Naples, Italy; c Cardiovascular Research Unit, IRCCS MultiMedica, 20138, Milan, Italy; d Complex Operative Unity of Virology and Microbiology, University Hospital of Campania “Luigi Vanvitelli", 80138, Naples, Italy; e Clinical Pathology and Microbiology Unit, San Giovanni di Dio e Ruggi D'Aragona University Hospital, 84126, Salerno, Italy

**Keywords:** *Klebsiella pneumoniae*, Outer membrane vesicles, Virulence, Apoptosis, Host-pathogen interaction, Endoplasmic reticulum stress

## Abstract

The programmed cell death pathways of apoptosis are important in mammalian cellular protection from infections. The activation of these pathways depends on the presence of membrane receptors that bind bacterial components to activate the transduction mechanism. In addition to bacteria, these mechanisms can be activated by outer membrane vesicles (OMVs). OMVs are spherical vesicles of 20–250 nm diameter, constitutively released by Gram-negative bacteria. They contain several bacterial determinants including proteins, DNA/RNA and proteins, that activate different cellular processes in host cells. This study focused on *Klebsiella pneumoniae*-OMVs in activating death mechanisms in human bronchial epithelial cells (BEAS-2B). Characterization of purified OMVs was achieved by scanning electron microscopy, nanoparticle tracking analysis and protein profiling. Cell viability was assessed by the 3-(4,5-dimethylthiazol-2-yl)-2,5-diphenyltetrazolium bromide assay while apoptotic induction was measured by flow cytometry and confirmed by western blotting. The OMVs produced showed a spherical morphology, with a diameter of 137.2 ± 41 nm and a vesicular density of 7.8 × 10^9^ particles/mL Exposure of cell monolayers to 50 μg of *K. pneumoniae*-OMV for 14 h resulted in approximately 25 % cytotoxicity and 41.15–41.14 % of cells undergoing early and late apoptosis. Fluorescence microscopy revealed reduced cellular density, the presence of apoptotic bodies, chromatin condensation, and nuclear membrane blebbing in residual cells. Activation of caspases −3 and −9 and dysregulation of BAX, BIM and Bcl-xL indicated the activation of mitochondria-dependent apoptosis. Furthermore, a decrease in the antioxidant enzymes superoxide dismutase, catalase and glutathione peroxidase involved endoplasmic reticulum stress with the potential formation of reactive oxygen species. These findings provide evidence for the role of OMVs in apoptosis and involvement in the pathogenesis of *K. pneumoniae* infections.

## Highlights

• *Klebsiella pneumoniae* is a worrying opportunistic pathogen in healthcare, responsible for multidrug-resistant infections.
• *Klebsiella pneumoniae* releases Outer Membrane Vesicles (20–250 nm), containing proteins, lipids and nucleic acids.
• Outer Membrane Vesicles perform several functions and contribute to augmented bacterial pathogenesis.
• Outer Membrane Vesicles in host-pathogen interaction induce mitochondrion-dependent intrinsic apoptosis and endoplasmic reticulum stress.

## 1 Introduction

*Klebsiella pneumoniae* (*K. pneumo*niae) is an opportunistic pathogen, mainly associated with community-acquired and nosocomial infections [1,2]. It is responsible for a wide spectrum of diseases such as bloodstream, lung, urinary tract, abdominal cavity, and soft tissue infections [3,4]. The high virulence of *K. pneumoniae* is attributed to the presence of lipopolysaccharide (LPS), capsule, adhesins and siderophores, necessary for colonization, adherence, invasion, and progression of the infection [5,6]. Furthermore, the rapid development of antimicrobial resistance compromises the choice of an adequate drug therapy to combat multidrug-resistant (MDR) *K. pneumoniae* infections [7]. Therefore, these infections involve high morbidity and mortality rates [8]. For instance, carbapenem-resistant *K. pneumoniae* diseases increased rapidly in China in 2020 due to the extensive use of carbapenems, dramatically limiting treatment options for managing infections [9]. This dramatic scenario makes it necessary to broaden the understanding of pathogenic mechanisms to establish new alternative strategies for treating and controlling *K. pneumoniae* infections [6].

The propensity of Gram-negative bacteria to constitutively release outer membrane vesicles (OMVs) during bacterial growth is now known [[10], [11], [12]]. OMVs are spherical nanostructures sized between 50 and 250 nm, secreted following swelling and budding of the outer membrane [13,14]. Purification and characterization techniques revealed the lipidic nature of these vesicles along with the presence of membrane, periplasmic and cytoplasmic proteins and nucleic acids (DNA and RNA) [14]. They have significant impacts in bacterial pathogenesis, contributing to inflammatory processes, tissue damage and immune evasion [15]. However, the mechanisms triggered by OMVs are to date incompletely understood [16]. Our previous studies identified the effect of OMVs on the miRNA expression profile in human bronchial epithelial cells (BEAS-2B), demonstrating upregulation and downregulation of 81 and 13 miRNA sequences, respectively, after 6 h of exposure with 10 μg of OMVs-derived *K. pneumoniae*. Among these, the sequences miR-223, hsa-miR-21, hsa-miR-25 and hsa-let-7g were validated, proving the modulation of inflammatory cytokine expression [17]. Another study confirmed the inflammasome activation, through the release of Interleukin (IL)-8, IL-6, IL-1β, and tumor necrosis factor (TNF)-α from BEAS-2B cells, after stimulation with 5 μg of OMVs [18]. Despite these advances, the role of *K. pneumoniae*-OMVs in modulating cell death signaling pathways remains unknown.

Toxins commonly present in *Escherichia coli* (*E. coli*) and *Neisseria gonorrhoeae* (*N. gonorrhoeae*) -OMVs, such as hemolysin and outer membrane protein (Por)-B are known to activate mitochondrial apoptosis, triggering the release of cytochrome *c*. The latter, in turn, activates the initiator caspase-9, caspase-3 and caspase-7 effectors, inducing cell death [19]. Bielaszewska et al. reported that the enterohemorrhagic hemolysin (EHEC-Hly) present in OMVs was internalized in human colorectal adenocarcinoma (Caco-2) cells via dynamin-dependent endocytosis mechanism. The vesicles were translocated into the endo-lysosomal compartments and the toxin targeted the mitochondria, causing a decrease in the mitochondrial transmembrane potential and the translocation of cytochrome *c* into the cytosol [20]. Furthermore, Deo et al. identified, for the first time, that after exposure to OMVs derived from *N. gonorrhoeae*, uropathogenic *E. coli* and *Pseudomonas aeruginosa* macrophages detect mitochondrial stress. Mitochondrial function alterations induced by exposure to OMVs activates Bcl-2-associated X (BAX), (B-cell lymphoma 2) BCL-2 and Myeloid cell leukemia-1 (MCL-1) resulting in protein synthesis inhibition and parallel initiation of caspase-11-mediated pyroptosis, culminating in the activation of the nucleotide-binding domain, leucine-rich–containing family, pyrin domain–containing-3 (NLRP3) inflammasome [21].

Contextually, our study investigated the impact of *K. pneumoniae*-OMVs on BEAS-2B cell death signaling pathways activation. Specifically, mitochondrial dysfunction was identified through the regulation of BAX, B-cell lymphoma-2 extra-large (Bcl-xL) and Bcl-2 Interacting Mediator of cell death (BIM) proteins, with simultaneous activation of initiator caspase-9 and caspase-3 effectors. The results indicated the possibility of apoptosome formation upon the OMVs contact with the host cell, highlighting their contribution to *K. pneumoniae* pathogenesis.

## 2 Material and methods

### 2.1 Bacterial strain and cell cultures

*K. pneumoniae* strain PCI 602 (ATCC 10031) was purchased from the American Type Culture Collection (ATCC, Manassas, VA, USA). Bacteria were cultured on Luria Bertani (LB)-agar plates to allow colony growth and in LB-broth for OMVs production. Human Bronchial Epithelial cell line (BEAS-2B CRL-9609) was purchased from ATCC and cultured in Dulbecco's Modified Eagle Medium/Nutrient Mixture F-12 (DMEM/F12; Gibco; Thermo Fisher Scientific, Waltham, MA, USA) supplemented with 10 % fetal bovine serum (FBS; Gibco; Thermo Fisher Scientific, Waltham, MA, USA) in a humidified atmosphere with 5 % CO_2_ at 37 °C.

### 2.2 OMVs purification

OMVs were isolated following a modified protocol from our previous studies. In detail, *K. pneumoniae* was grown in LB-broth (2 L, 37 °C, 180 rpm) until reaching the optical density (OD) 600 nm = 1, which corresponds to the end of the exponential growth curve. Then the bacterial suspension was centrifuged (4000×*g* for 20 min at 4 °C) to remove cells and the supernatant was filtered using vacuum Stericup™ 0.45 μm and at 0.22 μm pore size polyethersulfone (PES) top filter (Millipore, Burlington, MA, USA). The filtered supernatant (100 μL) was seeded on LB-agar plates to confirm the bacteria absence and concentrated to a final volume of 25 mL using Amicon® Stirred Cells (Millipore, Burlington, MA, USA) with a Millipore YM30 membrane (cut-off 30 kDa, Millipore, Burlington, MA, USA). The vesicular suspension was then centrifuged at 185.000×*g* for 2.5 h at 4 °C (Beckman Coulter Optima XPN-100 centrifuge and SW 32 Ti Rotor, Palo Alto, CA, USA), the pellet was washed in sterile 1 × Phosphate-buffered saline (1 × PBS) by ultracentrifugation and resuspended in 200 μL of 1 × PBS. To verify the sterility of the sample, 10 μL of vesicles were plated on LB-agar plates, incubated overnight (ON) at 37 °C. The purified OMVs were stored at −20 °C until use.

### 2.3 Nanoparticle tracking analysis (NTA)

The OMVs concentration and size were determined using a NanoSight NS300 nanoparticle tracking instrument (NTA) (Malvern Panalytical, Worcestershire, UK). OMV samples were diluted 1/100 in 1 × PBS buffer pH 7.4. Samples were infused into the NanoSight instrument using a syringe pump at a speed setting of “40” and a camera level of “10”. A total of 5 readings lasting 60 s each were acquired at room temperature (20.7–20.9 °C). A number of 1498 frames per sample were analyzed with NTA software (Malvern Instruments, version 3.2, Worcestershire, UK) with a detection threshold of 5. The mean size (nm), the mode (nm) and the concentration (particles/mL) were generated at the end of the analysis.

### 2.4 Transmission electron microscopy (TEM)

The isolated OMVs were visualized by TEM, through negative staining. The sample was diluted 1/10 in 1 × PBS and 5 μL was adsorbed onto carbon-coated copper/palladium grids for 30 min. To remove excess vesicles, the grids were washed with a drop of sterile deionized water. Then, negative staining was performed by adding 5 μL of 1 % (w/v) uranyl acetate. TEM images were acquired using an EM 208 S transmission electron microscope (Philips, Amsterdam, Netherlands).

### 2.5 Cytotoxicity profile

The OMVs cytotoxicity on BEAS-2B cells was determined using the 3-(4,5-dimethylthiazol-2-yl)-2,5-diphenyltetrazolium bromide (MTT) assay. Cells were seeded at a density of 2 × 10^4^ cells/well in 96-well microtiter plates and incubated at 37 °C with 5 % CO_2_, ON. Then, cells were exposed to OMVs (50–1 μg) for 6 and 14 h. Cells treated with the solvent used to dissolve and resuspend the OMVs (1 × PBS) represented the negative control (CTRL−), while cells treated with DMSO (100 %) constituted the positive control (CTRL+). After incubation, 100 μL of MTT solution (Sigma-Aldrich, St. Louis, MO, USA) (0.3 mg/mL) was added to each well for 3 h at 37 °C. The solution was removed and the formazan crystals were solubilized by adding 100 μL of DMSO (100 %). The absorbance at 570 nm was measured using a microplate reader (Tecan, Männedorf, Swiss) and the percentage of cell viability was calculated by the following formula:

% Cell viability = [100 × (*OD*570 nm *of t*ℎ*e test sampl*e/ O*D*570 nm *of CTR*−)]

### 2.6 Cellular exposure to OMVs

BEAS-2B cells (5 × 10^5^ cells per well) were plated in a 6-well plate and incubated ON at 37 °C with 5 % CO_2_. Then, the culture medium was removed and the cells were exposed to OMVs (50 μg) for 6 and 14 h. The same volume of OMVs solvent (1 × PBS) was added to the cells as CTRL-, while the cells exposed to LPS (50 μg) were used as CTRL+. After the treatment, the cells were harvested and subjected to subsequent analyses.

### 2.7 Flow cytometry

The apoptotic role of OMVs in BEAS-2B was investigated using the Annexin-V-Binding Buffer, according to the manufacturer's protocol (BioLegend, Cat. No. 422201, California, USA). After treatment, cells were detached with a scraper and washed twice with a cold BioLegend cell staining buffer. The suspension was resuspended in Annexin V Binding Buffer at 1 × 10^6^ cells/mL concentration. A volume of 100 μL was incubated with 5 μL of fluorochrome-conjugated Annexin V and PI, respectively. The samples were gently vortexed and incubated for 15 min at room temperature (25 °C) in the dark. Then 400 μL of annexin V binding buffer was added to each tube and analyzed using FACSVerse Flow Cytometer (BD Biosciences, San Francisco, USA). The gating strategy used to assess apoptotic potential included the analysis of the entire harvested cell population (Supplementary Fig. 1).

### 2.8 Western blotting analysis

After vesicular exposure, cells were harvested and lysed in RIPA lysis buffer, for 1 h on ice. The lysate was centrifuged at 14.000×*g* for 30 min at 4 °C to remove the cell debris and proteins were quantified by Bradford assay (HIMEDIA, Einhausen, Germany). Approximately 20 μg of proteins were separated on 10 and 12 % SDS-PAGE at 90 V for 1 h and 120 V for 1 h and then transferred onto a nitrocellulose membrane. Then, blocking with a 5 % solution of milk powder dissolved in Tris-buffered saline containing 0.1 % Tween-20 (TBST) was conducted for 1 h at room temperature under orbital shaking. The membranes were incubated overnight with the following primary antibodies: eIF2α (Cell signaling technology #9721, Rabbit 1:1000), CHOP (Cell signaling technology #2895, Mouse mAb 1:1000), Caspase-3 (Cell signaling technology #9662, Rabbit 1:1000), Caspase-3 (Santa Cruz #7148, Rabbit pAb 1:1000), BIM (Cell signaling technology #2933, Rabbit mAb 1:1000), Phospho-AKT1 (Invitrogen, #700392, Rabbit mAb 1:1000), NFκB p65 (Santa Cruz #8008, Mouse mAb 1:1000), SOD-1 (Cell signaling technology #4266, Mouse mAb 1:1000), BAX (Cell signaling technology #5023, Rabbit mAb 1:1000), Bcl-xL (Cell signaling technology #2764, Rabbit mAb 1:1000), Caspase-9 (Santa Cruz #8355, Rabbit pAb 1:1000), GPX-1 (Cell signaling technology #4266, Rabbit mAb 1:1000), CAT-1 (Sigma Aldrich #SAB2105074, Rabbit mAb 1:1000). Secondary anti-mouse and anti-rabbit antibodies (Bio-rad, Hercules, CA, USA) were used for the immunodetection of the investigated proteins, and blots were developed using enhanced chemiluminescence detection reagents (ECL, Thermofisher, Waltham, MA, USA), according to the manufacturer's instructions. Then, the signal was detected by exposure to X-ray films and the obtained data were analyzed using Image J software 1.48v (U.S. National Institutes of Health, Bethesda, MD, USA). The recorded optical density was expressed as a ratio relative to the GAPDH or β-actin proteins.

### 2.9 In situ cell death detection (TUNEL assay)

BEAS-2B cells were seeded as described in section 2.6 and treated with OMVs (50 μg/mL) and LPS (50 μg/mL) for 14 h. Cells from each treatment were fixed in 4 % paraformaldehyde and permeabilized in 0.25 % Triton-X 100. Detection of single-cell apoptosis was investigated using the Click-iT Plus TUNEL Assay, following the manufacturer's instructions (Invitrogen, Massachusetts, USA). The principle of the test involves the addition of modified EdUTPs by the enzyme terminal deoxynucleotidyl transferase (TdT) to the 3′-OH ends of fragmented DNA during the apoptotic process. Subsequently, the wells were counterstained with Hoechst 33342 for 15 min at room temperature in a dark environment (Invitrogen, Massachusetts, USA). Three random images were acquired for each well using the TCS SP5 confocal laser scanning microscope at 20 × magnification (Leica Microsystems, Wetzlar, Germany).

### 2.10 Statistical analysis

Three biological OMV replicates were obtained to conduct the experiments, which were performed in technical duplicates. The collected data were expressed as mean ± standard deviation (SD). The significance of the difference between the treated and CTRL-samples was obtained with Dunnett's test and ANOVA analysis, using The GraphPad Software Prism 9.0 (San Diego, CA, USA). The *p-value* <0.05 was considered significant.

## 3 Results

### 3.1 Characterization of *K. pneumoniae*-OMVs

**Fig. 1:**
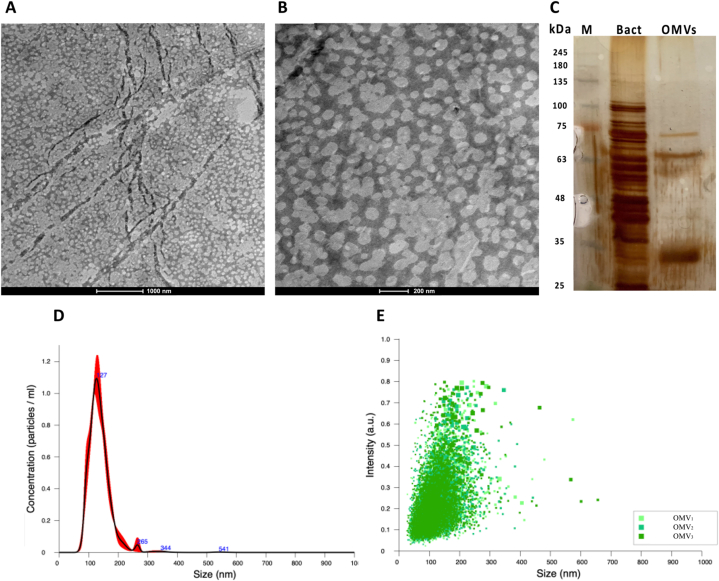
Characterization of OMVs derived from *K. pneumoniae* ATCC 10031. (A–B) TEM showed the presence of electron-dense vesicles with uniform spherical morphology and absence of bacterial contaminants; (C) Silver-stained SDS-PAGE (10 %) protein profiles of *K. pneumoniae* ATCC 10031 and related OMVs. The molecular mass marker (M) was expressed in kilodaltons (kDa); compared to the bacterial protein profile, only three bands at 75, 60 and 32 KDa were identified in OMVs. NTA analysis of OMVs: (D) FTLA (Finite Track Length Analysis) concentration/size graph, (E) Intensity/size graph. Concentration and mean hydrodynamic diameter indicated a mean vesicular diameter of 137.2 ± 41 nm and a concentration of 7.8 × 10^9^ particles/mL.

OMVs derived from *K. pneumoniae* ATCC 10031 were collected during late log phase growth from the clarified bacterial culture supernatant. TEM analysis showed a circular vesicle-like shape with a size of 20–200 nm and the absence of bacterial contaminants (Fig. 1A and B). NTA analysis was performed to better understand the diameter of the OMVs and the concentration. The latter recorded a medium vesicular diameter of ∼137.2 ± 41 nm, a mode value of 126.2 nm and a concentration of 7.8 × 10^9^ particles/mL, obtained from a starting volume of 2 L of bacterial culture (Fig. 1D–F). Then, 5 μg of OMVs were subjected to 10 % SDS-PAGE and visualized by silver nitrate staining. Three main bands, between 75 and 60 KDa and one band at about 32 KDa, were detected in OMVs from *K. pneumoniae* ATCC 10031, with a notable difference compared to the bacterial protein profile, which confirmed the absence of contaminants (Fig. 1 C). The results obtained are consistent with previous data showing the purification of OMVs from *K. pneumoniae* [17,18].

### 3.2 Exposure to *K. pneumoniae*-OMVs induces apoptosis in BEAS-2B cells

**Fig. 2:**
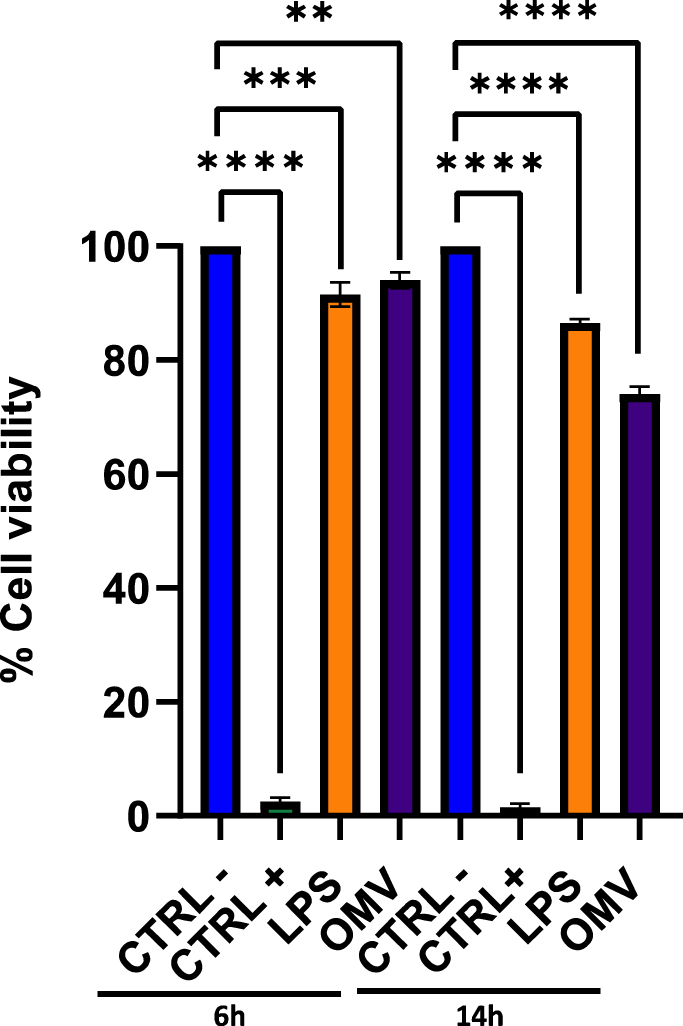
Cellular cytotoxicity assay performed via MTT assay. BEAS-2B cells were exposed to 50 μg of OMVs purified from *K*. *pneumoniae* ATCC 10031. Untreated cells represented CTRL-while cells treated with DMSO (100 %) corresponded to CTRL+. After 6 and 14 h of treatment, the cell monolayer was treated for 3 h with the MTT solution (0.3 mg/mL) and the formazan crystals were solubilized with 100 % DMSO. Cell viability was measured spectrophotometrically at 570 nm. After 14 h, a rate of 25 and 11.7 % cell death was recorded following exposure to *K. pneumoniae*-OMVs and LPS, respectively. The data represent the mean ± SD of 3 independent experiments. Dunnett's multiple comparisons tests: **: *p-value* = 0.0047; ***: *p-value* = 0.0004; ****: *p-value* < 0.0001.

BEAS-2B cells were exposed to 50 μg of OMVs for 6 and 14 h, followed by cell viability assay and cell apoptosis analysis performed by MTT and flow cytometry, respectively. The MTT assay indicated no relevant changes compared to unexposed cells in the first 6 h of treatment, recording 95 % ± 0.1 and 93 % ± 0.4 cell viability after treatment with vesicles and LPS, respectively; on the other hand, immediate complete cell death was determined by treating cells with 100 % DMSO, used as CTRL+. After 14 h of treatment, a 25 % increase in cell death induced by OMVs was verified, about 2.13 times more than exposure to LPS, which recorded a cytotoxicity rate of 11.7 % ± 0.7 (Fig. 2).

**Fig. 3:**
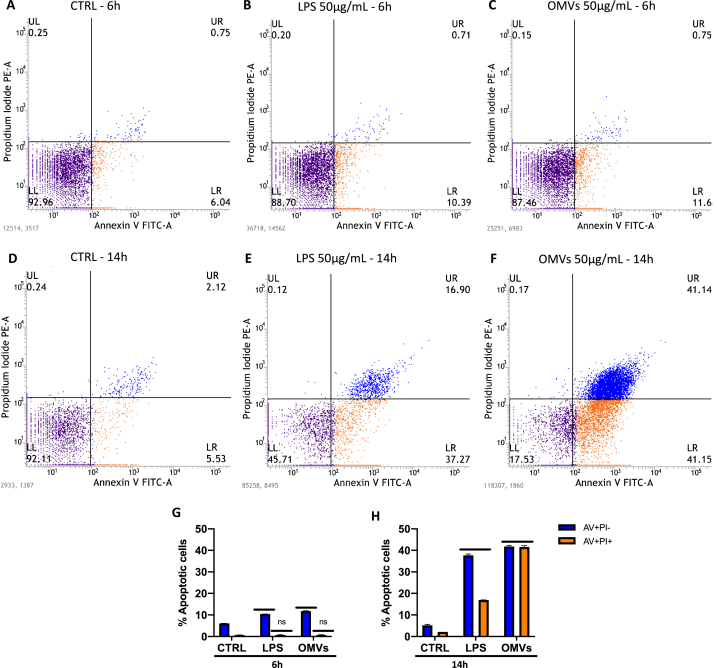
Flow cytometry analysis. BEAS-2B cells were exposed to 50 μg of *K. penumoniae*-OMVs for 6 (C) and 14 h (F). Untreated cells (A and D) and exposed to 50 μg of LPS (B and E) represented CTRL and CTRL+, respectively. After treatment, cells were detached and treated with fluorochrome-conjugated Annexin V and PI to evaluate cell damage. Dot plots were obtained by flow cytometry measuring PI/Annexin V fluorescence and the upper/lower right quadrants were used to measure apoptotic cells. Histograms indicate the total percentage of early (Annexin V-positive cells/PI-negative cells) and late (Annexin V/PI-double positive cells) apoptotic events after 6 (G) and 14 h (H). The 14-h treatment detected 41.15 and 41.14 % of early and late apoptosis, in contrast with LPS exposure which induced 37.27 and 16.90 % of early and late apoptosis. Unexposed cells recorded 5.53 and 2.12 % physiological apoptosis. The data represent the mean ± SD of 3 independent experiments. Dunnett's multiple comparisons tests: ns: *p-value* > 0.80; ****: *p-value* < 0.0001.

To elucidate the cell death mechanism involved, AnnexinV/Propidium Iodide staining and flow cytometric analysis was performed (Fig. 3A–H). As reported in representative dot plots, FACS analysis showed an increase in the percentage of cells in early (AV + PI-) and late (AV + PI+) apoptosis of 11.6 and 0.75 % respectively in cells exposed to OMVs and of 10.39 and 0.71 % in cells exposed to LPS in the first hours of treatment. In contrast, no relevant physiological death by apoptosis was recorded in the unexposed cells. After 14 h of exposure, a drastic increase in cell apoptosis death was recorded. In detail, 41.15 and 41.14 % of cells infected with OMVs underwent early and late apoptosis after LPS exposure cells detected in early and late apoptosis were respectively 37.27 and 16.90 % whereas 5.53 and 2.12 % of control cells were apoptotic.

### 3.3 Detection of apoptosis by Alexa Fluor and Hoechst staining

**Fig. 4:**
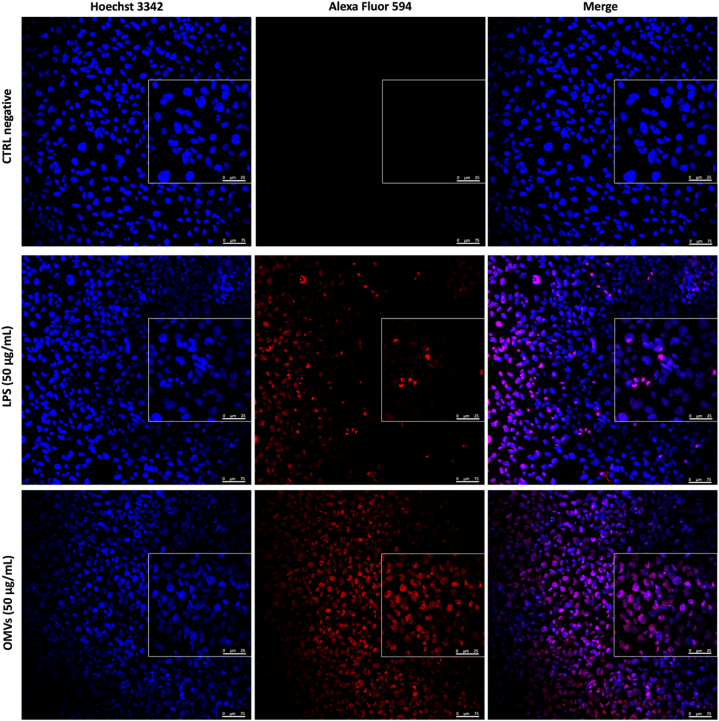
Detection of apoptosis by TUNEL assay. The cell monolayer exposed to *K. pneumoniae*-OMV (50 μg) and LPS (50 μg) for 14 h was fixed with 4 % paraformaldehyde and permeabilized with 0.25 % Triton-X 100. DNA fragmentation was detected through labeling of the 3′-OH terminals with EdUTP, mediated by the TdT enzyme. Images were acquired using the TCS SP5 laser scanning confocal microscope at 20 × magnification, with a 2.5× zoom of a representative field. The unexposed cells (CTRL negative) showed regular nuclei and uniform blue staining of the chromatin, while the cells treated with purified vesicles and LPS were TUNEL positive, recording an intense red staining. (For interpretation of the references to color in this figure legend, the reader is referred to the Web version of this article.)

To visualize the effect of OMVs on BEAS-2B cells after 14 h of treatment, cell monolayers were fixed in 4 % paraformaldehyde, permeabilized with 0.25 % Triton-X 100 and analyzed with the TUNEL Assay. Untreated cells, corresponding to CTRL negative, showed regular nuclei and uniform blue staining of chromatin using the dye Hoechst 3342, which binds adenine-thymine (AT) in the minor groove of DNA. Otherwise, BEAS-2B cells exposed to OMVs showed apoptotic bodies, chromatin condensation and nuclear membrane blebbing. Indeed, the intense red fluorescence demonstrated that the dye Alexa Fluor 594 specifically bounds the 3′-OH of fragmented DNA during the apoptosis processes. A similar trend occurred in response to LPS exposure, detecting red fluorescence, membrane irregularities and chromatin condensation, although less intense than the OMVs-treated sample (Fig. 4).

### 3.4 Western blot analysis of apoptotic target proteins

**Fig. 5:**
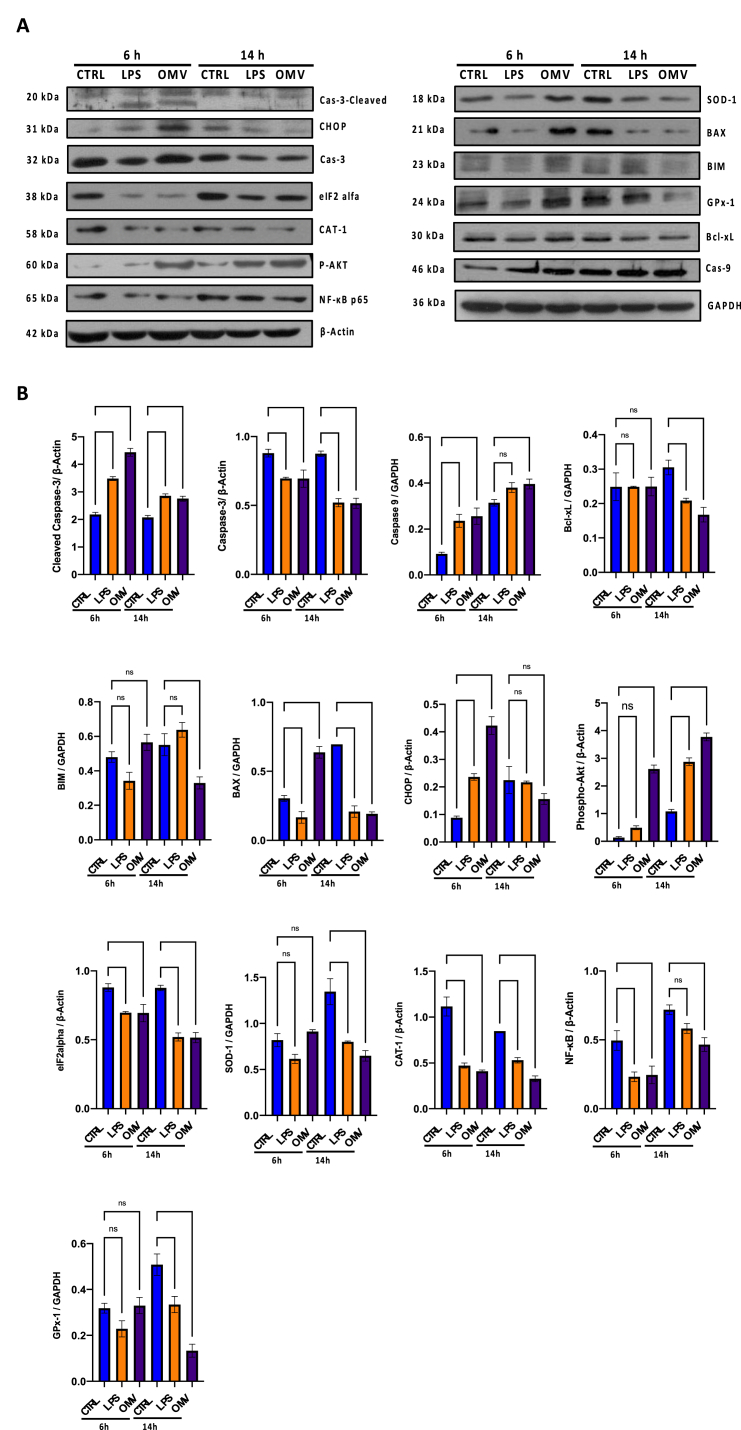
Western blot analysis for apoptosis-related proteins in BEAS-2B cells treated with OMVs and LPS for 6 and 14 h. **A**) All Western blot data were analyzed from the two or three independent experiments, and the bands' intensity was analyzed using Image J software 1.48v. The data were expressed as a ratio relative to the GAPDH or β-actin proteins. B) analysis of the main apoptosis marker proteins: Caspase-3 cleaved (**: *p-value* = 0.0011, ***: *p-value* = 0.0005, ****: *p-value* < 0.0001); Caspase 3 (**: *p-value* < 0.0079, ***: *p-value* < 0.0002); Caspase 9 (ns: *p-value* = 0.155, *: *p-value* = 0.0219, **: *p-value* < 0.0022); Bcl-xL (ns: *p-value* > 0.999, *: *p-value* = 0.0251, **: *p-value* = 0.0044); BIM (ns: *p-value* > 0.377, *: *p-value* = 0.0117); BAX (*: *p-value* < 0.0424, ***: *p-value* = 0.0002, ****: *p-value* < 0.0001); CHOP (ns: *p-value* = 0.145, **: *p-value* < 0.0050, ****: *p-value* < 0.0001); Phospho-AKT1 (ns: *p-value* = 0.672, ****: *p-value* < 0.0001); eIF2α (**: *p-value* < 0.0079, ***: *p-value* = 0.0002); SOD-1 (ns: *p-value* > 0.677, **: *p-value* = 0.0011, ***: *p-value* = 0.0003); CAT-1 (**: *p-value* = 0.0022, ***: *p-value* = 0.0001, ****: *p-value* < 0.0001); NFκB p65 (ns: *p-value* = 0.1357, *: *p-value* = 0.0103, **: *p-value* < 0.0097); GPX-1 (ns: *p-value* > 0.997, **: *p-value* = 0.0096, ***: *p-value* = 0.0001). The original images are shown in Supplementary Fig. 2.

Based on the results obtained by flow cytometry, we evaluated the expression of apoptosis-related proteins. Caspase-9, Caspase-3, BAX, Bcl-xL and BIM were investigated by western-blot analysis. The increase in immunoreactivity of Caspase-9 was already evident after only 6 h of treatment with OMVs and LPS, recording a fold-increase of 3.24 and 2.95, compared to unexposed cells. Meanwhile, Caspase-3 expression was reduced as a consequence of both treatments by approximately 1.38-fold compared with the control group, with an increase in cleavage of p12 and p17 subunits to form the active caspase-3 enzyme. Then the mitochondrial proteins BAX, Bcl-xL, BIM and NFκB were examined. Surprisingly, the Bcl-xL enzyme did not show any significant changes in the first 6 h of treatment, but a 1.75- and 1.57-fold reduction was recorded following OMVs and LPS treatment for 14 h. Instead, a 2.1- and 1.3-fold increase in the proapoptotic proteins BAX and BIM was detected after treatment with OMVs for 6 h, while no variation occurred with LPS exposure; lastly, a 2.5 and 2.7-fold reduction in NFκB was noticed in OMVs and LPS cells treated for 6 h. These data are consistent with the previously described flow cytometry analysis, highlighting the activation of the mitochondrial apoptotic signaling pathway. To further explore the mechanism involved in cellular apoptosis, the expression levels of specific endoplasmic reticulum stress markers, including CHOP, Phospho-AKT1, and eIF2α were analyzed. OMVs and LPS induced a 5.3 and 2.9-fold increase in CHOP expression levels, with a parallel increase of 3.7 and 2.8 of Phospho-AKT1 in the 14 h of treatment; conversely, the expression levels of the eIF2α protein showed a gradual decrease during the 14 h of treatment, respect to cell group. As confirmation of the induction of oxidative stress, SOD-1 CAT-1 and GPX-1 levels were also investigated. All three markers showed a significant reduction in response to 14 h of treatment. Specifically, a 2.3-1.8, 2.4-1.6 and 4.1–1.7-fold decrease of SOD-1, CAT-1 e GPX-1 was recorded after exposure to OMVs and LPS (Fig. 5A and B).

## 4 Discussion

*K. pneumoniae* is an opportunistic pathogen responsible for worrying infections in healthcare settings that compromise and threaten human health [22,23]. This strain produces and releases OMVs, which are recognized to play different roles in bacterial physiology and pathogenesis [24]. Previous studies have shown that OMVs evade the immune system by masking the bacterial components and reducing recognition by host immune cells [25,26]. They also increase virulence by contributing to biofilm formation and promoting bacterial survival and proliferation in the host [11]. Moreover, vesicles can bind and interact with host cells, promoting the establishment of chronic inflammation and modulating the host immune response through the release of cytokines and chemokines [27].

**Fig. 6:**
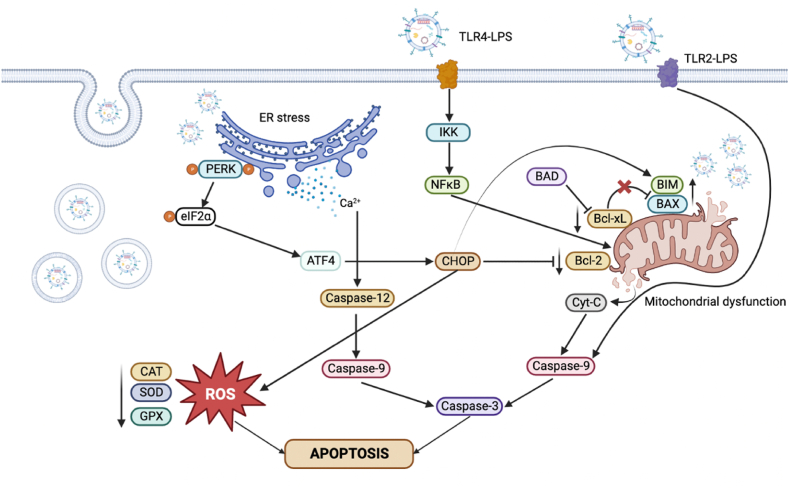
Schematic representation of apoptosis signalling pathways triggered by *K. pneumoniae*-OMVs in host cells.

To date, a poorly studied aspect of the host-pathogen interactions mediated by *K. pneumoniae-*OMVs is the triggering of programmed cell death processes. Apoptosis is a vital phenomenon in all multicellular organisms and plays a key role in maintaining homeostasis and tissue development [28,29]. It is regulated by several stimuli including growth factor deprivation, DNA damage and oxidative stress, which activate cellular signaling pathways [30]. OMVs derived from several bacterial species have been shown to induce apoptosis in numerous cell lines, such as epithelial, endothelial, and immune cells [19,31]. Nevertheless, OMVs-mediated apoptosis is not fully understood but is thought to be activated by specific receptors on the surface of target cells, thereby catalyzing the signaling pathways that lead to apoptosis [16]. A proposed mechanism of OMV-induced apoptosis identified toll-like receptor (TLR) 4, as a key inducer of apoptotic signal transduction [32]. A second advanced mechanism reported the release of pro-apoptotic factors (cytokines and enzymes) into the surrounding environment following the cellular internalization of vesicles [16]. Indeed, it has been proved that the endocytosis of *P. aeruginosa* OMVs involves apoptosis in host cells by the enzyme cytotoxic necrotizing factor (CNF1), which cleaves the cytoskeletal protein actin [33]. The limited evidence available pushes us to further detail the causes and mechanisms of programmed cell death induced by *K. pneumoniae*-OMVs. Therefore, the vesicles were purified and characterized in terms of morphology, density and protein profile by TEM, NTA and SDS-PAGE. The vesicles appeared to have a spherical morphology with a diameter of 137.2 ± 41 nm and a less complex protein profile than that of the producing bacterial strain [34]. Moreover, the vesicular suspension obtained from 2 L of stationary phase culture was estimated to be 7.8 × 10^9^ particles/mL. Thereafter, bronchial epithelial cells were exposed for 6 and 14 h to *K. pneumoniae*-OMVs. In a time-dependent manner, 50 μg of OMVs induced an increase in early and late apoptosis, which was barely detected by the lower sensitivity MTT cytotoxic assay, revealing only cytotoxicity. The western-blotting analysis demonstrated the activation of initiator caspases −3 and −9 and the dysregulation of mitochondrial proteins BAX, Bcl-xL and BIM. Considering our results, a potential process primed by OMVs was the mitochondrion-dependent intrinsic apoptotic pathway, altering the balance between pro- and anti-apoptotic factors (Fig. 6). Consistently, Bielaszewska et al. demonstrated the activation of the intrinsic apoptotic pathway in human intestinal epithelial cells (Caco-2), brain microvascular endothelial cells (HBMEC) and renal glomerular endothelial cells (HRGEC), mediated by EHEC *E. coli*-OMVs. Internalization of vesicles and their contents including Shiga toxin 2a (Stx2a), cytolethal toxin V (CdtV), hemolysin EHEC and flagellin caused caspase-9 activation after 48 h of exposure [35]. Similarly, *N. gonorrhoeae*-OMVs induced mitochondrial apoptosis, leading to DNA degradation and consequently loss of cell viability. In detail, Deo et al. demonstrated that PorB located in the outer membrane of OMVs was able to penetrate macrophages and reach mitochondria, inducing cytochrome *c* release, caspase-3 activation, plasma membrane collapse and subsequent macrophages death [21]. OMV exposure resulted in early induction of the pro-apoptotic factors BAX and BIM, whose expression is markedly reduced after 14 h of vesicular stimulation. BAX is a pro-apoptotic factor, required for the permeabilization of the outer mitochondrial membrane during the apoptotic process. BAX factors assemble into large homo-oligomers to form channels in the mitochondrial membrane, releasing apoptogenic factors into the cytosol to trigger the apoptotic cascade. BIM activates BAX homo-oligomerization after approximately 30 min and continues over time, increasing the formation of membrane pores [36]. Through time-lapse imaging of single cells, Deo et al. demonstrated that exposure to *N. gonorrhoeae*-OMVs induced loss of mitochondrial membrane potential within 10 h [21]. In line with this evidence, in the present study, a high expression of the proapoptotic factors BAX and BIM was already recorded after 6 h of *K. pneumoniae*-OMVs exposure. Meanwhile, caspase 3 activation results in the degradation of proapoptotic molecules via ubiquitin/proteasome pathways [37]. This could explain the BIM/BAX reduction in the late stage of the infection. Deo et al. reported caspase-3 activation during 15 h of stimulation with vesicles derived from *N. gonorrhoeae* [19]. In agreement with our results, it has been demonstrated that the release of the cytochrome *c* in cytoplasm participates in the activation of caspase-9 which increases over time [38]. Otherwise, the late anti-apoptotic regulator Bcl-xL expression remains reduced up to 14 h of vesicular treatment. Bcl-xL maintained mitochondrial integrity, preventing the release of cytochrome *c* and caspase activation [39]. Consistent with the results described by Deo et al., Bcl-xL levels were initially elevated but gradually decreased over 24 h of exposure to *N. gonorrhoeae*-OMVs [19].

Beyond the mitochondrial impairment, our study highlighted the endoplasmic reticulum (ER) stress induction after vesicular exposure. Indeed, OMVs caused an increase in the expression of the pro-apoptotic C/EBP-homologous protein (CHOP) and a decrease in the activity of the antioxidant enzymes catalase, superoxide dismutase and glutathione peroxidase, leading to the burst of oxidative damage. Early modulation of eukaryotic initiation factor 2 subunit 1 (eIF2α) phosphorylation in OMV-exposed cells further corroborated a mechanism of ER stress-induced apoptosis Similarly, evidence reported that *Borrelia burgdorferi* (*B. burgdorferi*) -OMVs caused a significant increase in monocyte chemoattractant proteins 1 and -2 (MCP-1 and MCP-2), causing oxidative stress in neuronal cells. In response to 30 min of treatment with *B. burgdorferi*-OMVs, cellular levels of superoxide dismutase were reduced, remaining low for up to 24 h [40]. Overall, the data revealed that OMVs cause cellular stress conditions. Induction of cell death mechanisms induced by *K. pneumoniae*-OMVs could increase generalized tissue damage and inflammation. Although not fully clarified, currently the understanding of OMVs interaction with host cells is based on several hypotheses: i) charge interactions, since the outer membrane of Gram-negative bacteria and the membranes of eukaryotic cells have electrostatic charges different. This difference could facilitate the first contact between OMVs and host cells [27]; ii) adhesive proteins transported on the vesicle surface that could bind specific receptors or molecules on the host cell membrane and activate intracellular signal transduction pathways [41]; iii) endocytosis clathrin, caveolae, or dynamin -dependent [16]; iv) macropinocytosis, in which cells could phagocytose OMVs forming macrovesicles [16]. After internalization, OMVs release their cargo into the host cell cytoplasm or endosomal compartments, leading to the activation of intracellular signaling pathways (Fig. 6). OMVs release toxins into the intracellular environment which determine mitochondrial damage, reduction of antiapoptotic proteins with parallel activation of pro-apoptotic factors, triggering caspase-3 and -9-based activation of apoptosomes. Several lines of evidence highlighted the involvement of OMV-associated molecules in driving the processes underlying apoptosis. Tiku et al. demonstrated that the outer membrane protein A found in OMVs was internalized by the host cell, triggering the activation of the host GTPase dynamin-related protein 1 (DRP1). This, in turn, led to the accumulation of protein A on mitochondria, causing alterations in mitochondrial function, increased ROS, and ultimately apoptosis [42]. In the study conducted by Bielaszewska et al. hemolysin present in OMVs of enterohemorrhagic *E. coli* was confirmed to play a role in inducing apoptosis in HBMEC and Caco-2 cells. In these cellular models, vesicles were taken up via dynamin-dependent endocytosis. The acidification of endosomes and the resulting decrease in pH triggered the cleavage of the toxin from the vesicles, leading to its release, probably through its pore-forming activity. Hemolysin caused mitochondrial dysfunction, DNA fragmentation, and chromatin condensation, characteristic signs of programmed cell death [20]. Furthermore, Deo et al. reported that the PorB protein was implicated in inducing programmed death in macrophages. Translocation of PorB into mitochondria and dissipation of mitochondrial membrane potential resulted in a subsequent release of cytochrome *c* and activation of intrinsic apoptosis [19]. The results obtained and the evidence in the literature highlight the important OMVs implications in bacterial pathogenesis. However, limitations associated with the present study should be considered, which although it provides the first comprehensive understanding of *released K. pneumoniae*-induced cell apoptosis OMVs, did not investigate two important factors, which will be addressed in future studies: i) understanding the mechanism of OMVs binding to membrane receptors and/or the vesicles intracellular internalization, ii) define the OMVs dose/time-effect correlation of necessary to trigger cell death mechanisms.

## Data availability statement

The data that support the findings of this study are available from the corresponding author (gfranci@unisa.it) upon reasonable request.

## Funding statement

We used external funds from "Univerity Founds for basic research 2022, code: ORSA223933"

## CRediT authorship contribution statement

**Federica Dell'Annunziata:** Conceptualization. **Elena Ciaglia:** Data curation. **Veronica Folliero:** Formal analysis. **Valentina Lopardo:** Investigation. **Anna Maciag:** Methodology. **Massimiliano Galdiero:** Validation. **Annibale Alessandro Puca:** Visualization. **Gianluigi Franci:** Funding acquisition.

## Declaration of competing interest

The authors declare that they have no known competing financial interests or personal relationships that could have appeared to influence the work reported in this paper.

## References

[1] Martin R.M., Bachman M.A. (2018). Colonization, infection, and the accessory genome of Klebsiella pneumoniae. Front. Cell. Infect. Microbiol..

[2] Navon-Venezia S., Kondratyeva K., Carattoli A. (2017). Klebsiella pneumoniae: a major worldwide source and shuttle for antibiotic resistance. FEMS Microbiol. Rev..

[3] Paczosa M.K., Mecsas J. (2016). Klebsiella pneumoniae: going on the offense with a strong defense. Microbiol. Mol. Biol. Rev..

[4] Awoke T., Teka B., Seman A., Sebre S., Yeshitela B., Aseffa A., Mihret A., Abebe T. (2021). High prevalence of multidrug-resistant Klebsiella pneumoniae in a tertiary care hospital in Ethiopia. Antibiotics (Basel).

[5] Assoni L., Girardello R., Converso T.R., Darrieux M. (2021). Current stage in the development of Klebsiella pneumoniae vaccines. Infect. Dis. Ther..

[6] Bengoechea J.A., Sa Pessoa J. (2019). Klebsiella pneumoniae infection biology: living to counteract host defences. FEMS Microbiol. Rev..

[7] Lopes E., Saavedra M.J., Costa E., de Lencastre H., Poirel L., Aires-de-Sousa M. (2020). Epidemiology of carbapenemase-producing Klebsiella pneumoniae in northern Portugal: predominance of KPC-2 and OXA-48. J Glob Antimicrob Resist.

[8] DeLeo F.R., Kobayashi S.D., Porter A.R., Freedman B., Dorward D.W., Chen L., Kreiswirth B.N. (2017). Survival of carbapenem-resistant Klebsiella pneumoniae sequence Type 258 in human blood. Antimicrob. Agents Chemother..

[9] Tesfa T., Mitiku H., Edae M., Assefa N. (2022). Prevalence and incidence of carbapenem-resistant K. pneumoniae colonization: systematic review and meta-analysis. Syst. Rev..

[10] Jan A.T. (2017). Outer membrane vesicles (OMVs) of gram-negative bacteria: a perspective update. Front. Microbiol..

[11] Furuyama N., Sircili M.P. (2021). Outer membrane vesicles (OMVs) produced by gram-negative bacteria: structure, functions, biogenesis, and vaccine application. BioMed Res. Int..

[12] Berleman J., Auer M. (2013). The role of bacterial outer membrane vesicles for intra- and interspecies delivery. Environ. Microbiol..

[13] Dell'Annunziata F., Folliero V., Giugliano R., De Filippis A., Santarcangelo C., Izzo V., Daglia M., Galdiero M., Arciola C.R., Franci G. (2021). Gene transfer potential of outer membrane vesicles of gram-negative bacteria. Int. J. Mol. Sci..

[14] Avila-Calderón E.D., Ruiz-Palma M.D.S., Aguilera-Arreola M.G., Velázquez-Guadarrama N., Ruiz E.A., Gomez-Lunar Z., Witonsky S., Contreras-Rodríguez A. (2021). Outer membrane vesicles of gram-negative bacteria: an outlook on biogenesis. Front. Microbiol..

[15] Villageliu D.N., Samuelson D.R. (2022). The role of bacterial membrane vesicles in human health and disease. Front. Microbiol..

[16] O'Donoghue E.J., Krachler A.M. (2016). Mechanisms of outer membrane vesicle entry into host cells. Cell Microbiol..

[17] Dell'Annunziata F., Ilisso C.P., Dell'Aversana C., Greco G., Coppola A., Martora F., Dal Piaz F., Donadio G., Falanga A., Galdiero M., Altucci L., Galdiero M., Porcelli M., Folliero V., Franci G. (2020). Outer membrane vesicles derived from Klebsiella pneumoniae influence the miRNA expression profile in human bronchial epithelial BEAS-2B cells. Microorganisms.

[18] Martora F., Pinto F., Folliero V., Cammarota M., Dell'Annunziata F., Squillaci G., Galdiero M., Morana A., Schiraldi C., Giovane A., Galdiero M., Franci G. (2019). Isolation, characterization and analysis of pro-inflammatory potential of Klebsiella pneumoniae outer membrane vesicles. Microb. Pathog..

[19] Deo P., Chow S.H., Hay I.D., Kleifeld O., Costin A., Elgass K.D., Jiang J.-H., Ramm G., Gabriel K., Dougan G., Lithgow T., Heinz E., Naderer T. (2018). Outer membrane vesicles from Neisseria gonorrhoeae target PorB to mitochondria and induce apoptosis. PLoS Pathog..

[20] Bielaszewska M., Marejková M., Bauwens A., Kunsmann-Prokscha L., Mellmann A., Karch H. (2018). Enterohemorrhagic Escherichia coli O157 outer membrane vesicles induce interleukin 8 production in human intestinal epithelial cells by signaling via Toll-like receptors TLR4 and TLR5 and activation of the nuclear factor NF-κB. Int J Med Microbiol.

[21] Deo P., Chow S.H., Han M.-L., Speir M., Huang C., Schittenhelm R.B., Dhital S., Emery J., Li J., Kile B.T., Vince J.E., Lawlor K.E., Naderer T. (2020). Mitochondrial dysfunction caused by outer membrane vesicles from Gram-negative bacteria activates intrinsic apoptosis and inflammation. Nat Microbiol.

[22] van Dorp L., Wang Q., Shaw L.P., Acman M., Brynildsrud O.B., Eldholm V., Wang R., Gao H., Yin Y., Chen H., Ding C., Farrer R.A., Didelot X., Balloux F., Wang H. (2019). Rapid phenotypic evolution in multidrug-resistant Klebsiella pneumoniae hospital outbreak strains. Microb. Genom..

[23] Mohd Asri N.A., Ahmad S., Mohamud R., Mohd Hanafi N., Mohd Zaidi N.F., Irekeola A.A., Shueb R.H., Yee L.C., Mohd Noor N., Mustafa F.H., Yean C.Y., Yusof N.Y. (2021). Global prevalence of nosocomial multidrug-resistant Klebsiella pneumoniae: a systematic review and meta-analysis. Antibiotics (Basel).

[24] Turner K.L., Cahill B.K., Dilello S.K., Gutel D., Brunson D.N., Albertí S., Ellis T.N. (2015). Porin loss impacts the host inflammatory response to outer membrane vesicles of Klebsiella pneumoniae. Antimicrob. Agents Chemother..

[25] Cai W., Kesavan D.K., Wan J., Abdelaziz M.H., Su Z., Xu H. (2018). Bacterial outer membrane vesicles, a potential vaccine candidate in interactions with host cells based. Diagn. Pathol..

[26] Mancini F., Rossi O., Necchi F., Micoli F. (2020). OMV vaccines and the role of TLR agonists in immune response. Int. J. Mol. Sci..

[27] Cecil J.D., Sirisaengtaksin N., O'Brien-Simpson N.M., Krachler A.M. (2019). Outer membrane vesicle-host cell interactions. Microbiol. Spectr..

[28] Elmore S. (2007). Apoptosis: a review of programmed cell death. Toxicol. Pathol..

[29] Singh R., Letai A., Sarosiek K. (2019). Regulation of apoptosis in health and disease: the balancing act of BCL-2 family proteins. Nat. Rev. Mol. Cell Biol..

[30] Renehan A.G., Booth C., Potten C.S. (2001). What is apoptosis, and why is it important?. BMJ.

[31] Rumbo C., Tomás M., Fernández Moreira E., Soares N.C., Carvajal M., Santillana E., Beceiro A., Romero A., Bou G. (2014). The Acinetobacter baumannii Omp33-36 porin is a virulence factor that induces apoptosis and modulates autophagy in human cells. Infect. Immun..

[32] Dhital S., Deo P., Stuart I., Naderer T. (2021). Bacterial outer membrane vesicles and host cell death signaling. Trends Microbiol..

[33] Chaoprasid P., Dersch P. (2021). The cytotoxic necrotizing factors (CNFs)-A family of rho GTPase-activating bacterial exotoxins. Toxins.

[34] Dell'Annunziata F., Dell'Aversana C., Doti N., Donadio G., Dal Piaz F., Izzo V., De Filippis A., Galdiero M., Altucci L., Boccia G., Galdiero M., Folliero V., Franci G. (2021). Outer membrane vesicles derived from Klebsiella pneumoniae are a driving force for horizontal gene transfer. Int. J. Mol. Sci..

[35] Bielaszewska M., Rüter C., Bauwens A., Greune L., Jarosch K.-A., Steil D., Zhang W., He X., Lloubes R., Fruth A., Kim K.S., Schmidt M.A., Dobrindt U., Mellmann A., Karch H. (2017). Host cell interactions of outer membrane vesicle-associated virulence factors of enterohemorrhagic Escherichia coli O157: intracellular delivery, trafficking and mechanisms of cell injury. PLoS Pathog..

[36] Cosentino K., Hertlein V., Jenner A., Dellmann T., Gojkovic M., Peña-Blanco A., Dadsena S., Wajngarten N., Danial J.S.H., Thevathasan J.V., Mund M., Ries J., Garcia-Saez A.J. (2022). The interplay between BAX and BAK tunes apoptotic pore growth to control mitochondrial-DNA-mediated inflammation. Mol Cell.

[37] Wakeyama H., Akiyama T., Takahashi K., Amano H., Kadono Y., Nakamura M., Oshima Y., Itabe H., Nakayama K.I., Nakayama K., Nakamura K., Tanaka S. (2007). Negative feedback loop in the Bim-caspase-3 axis regulating apoptosis and activity of osteoclasts. J. Bone Miner. Res..

[38] McDonnell M.A., Wang D., Khan S.M., Vander Heiden M.G., Kelekar A. (2003). Caspase-9 is activated in a cytochrome c-independent manner early during TNFalpha-induced apoptosis in murine cells. Cell Death Differ..

[39] Bleicken S., Wagner C., García-Sáez A.J. (2013). Mechanistic differences in the membrane activity of Bax and Bcl-xL correlate with their opposing roles in apoptosis. Biophys. J..

[40] Wawrzeniak K., Gaur G., Sapi E., Senejani A.G. (2020). Effect of Borrelia burgdorferi outer membrane vesicles on host oxidative stress response. Antibiotics (Basel).

[41] Bonnington K.E., Kuehn M.J. (2014). Protein selection and export via outer membrane vesicles. Biochim. Biophys. Acta.

[42] Tiku V., Kofoed E.M., Yan D., Kang J., Xu M., Reichelt M., Dikic I., Tan M.-W. (2021). Outer membrane vesicles containing OmpA induce mitochondrial fragmentation to promote pathogenesis of Acinetobacter baumannii. Sci. Rep..

