# Supplementary material for: Klebsiella pneumoniae-OMVs activate death-signaling pathways in Human Bronchial Epithelial Host Cells (BEAS-2B)
Source: Heliyon. 2024 Apr 13;10(8):e29017. doi: 10.1016/j.heliyon.2024.e29017 (PMC11031753; doi:10.1016/j.heliyon.2024.e29017)
Supplement: Multimedia component 1 [file mmc1.docx]

**Supplementary material**

**Supplementary Figure 1.** Cytometry gating strategy used to quantify the cell population undergoing early and late apoptosis after exposure to OMVs and LPS for 6 and 14 hours.

**Supplementary Figure 2.** Western blot analysis for apoptosis-related proteins in BEAS-2B cells treated with OMVs and LPS for 6 and 14 hours. Sample order: CTRL 6h - LPS 6h - OMV 6h, CTRL 14h - LPS 14h - OMV 14h. **A**) SOD-1, **B**) BAX, **C**) BIM, **D**) GPX-1, **E**) Bcl-xL, **F**) Caspase 9, **G)** GAPDH, **H**) Caspase-3 cleaved, **I**) CHOP, **J**) Caspase-3, **K**) eIF2α, **L**) CAT-1, **M**) Phospho-AKT1, **N**) NFκB p65, **O**) β-actin. **H**) and **B**) ponceau red gels
